# Single-Cell RNA Profiling of Human Skin Reveals Age-Related Loss of Dermal Sheath Cells and Their Contribution to a Juvenile Phenotype

**DOI:** 10.3389/fgene.2021.797747

**Published:** 2022-01-07

**Authors:** Juliane M. D. Ahlers, Cassandra Falckenhayn, Nicholas Holzscheck, Llorenç Solé-Boldo, Sabrina Schütz, Horst Wenck, Marc Winnefeld, Frank Lyko, Elke Grönniger, Annette Siracusa

**Affiliations:** ^1^ Beiersdorf AG, Research and Development, Hamburg, Germany; ^2^ Division of Epigenetics, DKFZ-ZMBH Alliance, German Cancer Research Center, Heidelberg, Germany

**Keywords:** single-cell RNA sequencing, skin, fibroblasts, dermal sheath cells, aging, stem cells, regeneration, Activin A

## Abstract

The dermal sheath (DS) is a population of mesenchyme-derived skin cells with emerging importance for skin homeostasis. The DS includes hair follicle dermal stem cells, which exhibit self-renewal and serve as bipotent progenitors of dermal papilla (DP) cells and DS cells. Upon aging, stem cells exhibit deficiencies in self-renewal and their number is reduced. While the DS of mice has been examined in considerable detail, our knowledge of the human DS, the pathways contributing to its self-renewal and differentiation capacity and potential paracrine effects important for tissue regeneration and aging is very limited. Using single-cell RNA sequencing of human skin biopsies from donors of different ages we have now analyzed the transcriptome of 72,048 cells, including 50,149 fibroblasts. Our results show that DS cells that exhibit stem cell characteristics were lost upon aging. We further show that *HES1*, *COL11A1*, *MYL4* and *CTNNB1* regulate DS stem cell characteristics. Finally, the DS secreted protein Activin A showed paracrine effects on keratinocytes and dermal fibroblasts, promoting proliferation, epidermal thickness and pro-collagen production. Our work provides a detailed description of human DS identity on the single-cell level, its loss upon aging, its stem cell characteristics and its contribution to a juvenile skin phenotype.

## Introduction

Upon aging, the phenotype of the skin changes and the epidermis becomes thinner (Farage et al., 2013). The deposition of extracellular matrix by the dermis is reduced (Varani et al., 2006) and its degradation is increased (Varani et al., 2000). While the epidermis is constantly renewed, damage in the dermis accumulates over time (Waldera-Lupa et al., 2014). Therefore, changes of the dermis are considered to make substantial contributions to the phenotype of aged skin.

Fibroblasts are major cellular constituents of the dermis. Two recent publications utilized single-cell RNA sequencing (scRNA-seq) to study age-related changes in human dermal fibroblasts (Solé-Boldo et al., 2020; Zou et al., 2021). Observations include a loss of fibroblast priming and the expression of skin aging-associated secreted proteins upon aging (Solé-Boldo et al., 2020), as well as a geroprotective effect of the *HES1* transcription factor (Zou et al., 2021). In another scRNA-seq study, it was hypothesized that minor fibroblast populations that possibly include only a few cells, may represent progenitors (Tabib et al., 2018). However, the identification and characterization of specific age-relevant human dermal fibroblast populations, e.g. mesenchymal progenitors, remains a crucial topic to understand skin aging and to develop tailored intervention strategies.

Stem cells are progenitors characterized by their differentiation potential, self-renewal and a stem cell secretome. They are particularly relevant for human tissue regeneration (Ayala-Cuellar et al., 2019) and aging (Lopez-Otin et al., 2013). While epithelial stem cells have been studied in considerable detail, very little is known about mesenchymal progenitors in the skin. The mesenchyme-derived dermal sheath (DS) of the hair follicle is thought to include hair follicle dermal stem cells (Rahmani et al., 2014). Most of our current knowledge of the DS, including hair follicle dermal stem cells, has been generated in mice using reporter constructs, lineage tracing and fluorescence-activated cell sorting. In the hair cycle, contraction of the DS is required for follicle regression. Therefore, the DS expresses the molecular machinery of smooth muscles e.g. smooth muscle-related genes *Acta2*, *Tagln*, *Myh11* and *Myl9* (Heitman et al., 2020). Although the mouse DS is remodeled during hair cycling, a subset of DS cells is retained in the dermal sheath cup (DSC) over consecutive hair cycles and these cells function as progenitors, giving rise to dermal papilla (DP) cells and DS cells (Rahmani et al., 2014). On the mechanistic level, Wnt signaling is involved in the self-renewal of hair follicle dermal stem cells, and their number was reported to be reduced upon aging (Shin et al., 2020).

In contrast to the mouse, our knowledge of the human DS is very limited. It has been described that *ACTA2* expression is conserved (Urabe et al., 1992). Furthermore, smooth muscle proteins TAGLN, MYH11 and to a lesser extent MYL9 were expressed in the DS of human scalp hair follicles (Heitman et al., 2020). Pioneering studies showed that isolated DS and DSC cells induce *de novo* hair follicle formation after transplantation or injection into human skin (Reynolds et al., 1999; Tsuboi et al., 2020). Based on in silico analyses, *TGFB1*, Wnt signalling molecules and BMP signalling antagonists have been suggested to be involved in the regulatory network of DSCs (Niiyama et al., 2018), but the functional roles of these candidate genes and pathways remain to be established. Furthermore, detailed knowledge of DS stem cell characteristics and paracrine effects on extrafollicular cell types including dermal fibroblasts and keratinocytes is still lacking, although it is crucial for understanding tissue regeneration and aging.

Using scRNA-seq, we have analyzed 72,048 skin cells, including 50,149 fibroblasts from whole skin biopsies of the outer forearm from three young (mean age 25.33) and four old (mean age 74.25) Caucasian female participants. Data analysis revealed a specific loss of DS cells with stem cell characteristics upon aging. We also observed that the genes *HES1*, *COL11A1*, *MYL4* and *CTNNB1* play an important role in regulating DS stem cell characteristics. DS-secreted proteins, especially Activin A, exhibited paracrine effects on keratinocytes and dermal fibroblasts, thus contributing to a juvenile skin phenotype.

## Materials and Methods

Detailed methods in supplementary methods.

### Study Participants

For scRNA-seq whole skin biopsies were obtained through clinical study approved by the Ethics Committee of the Medical Association of Hamburg (PV6054). For validation and functional experiments skin was purchased from Alphenyx, Marseille, France. A detailed overview of the participants and experiments, skin biopsies and cells is given in Supplementary Table S1.

### Droplet Based scRNA-Seq and Data Analysis

Droplet based scRNA-seq was done as previously described (Solé-Boldo et al., 2020). Clustering and cell type annotation was done using 10x Genomics Cell Ranger 2.1.0 (10x Genomics, Pleasanton, California) (Zheng et al., 2017) and Seurat package, version 3.6.4 (Stuart et al., 2019), in R, version 4.0.5 (R Core Team, 2020, https://www.R-project.org/). Cells with less than 200 or more than 7,500 expressed genes or cells expressing more than 5% mitochondrial reads were removed. RNA velocity was carried out as described (La Manno et al., 2018) using the velocyto. R package, version 0.6.

Stemness scores for individual cells were calculated using the significantly differentially expressed genes between human dermal fibroblasts and fibroblast-derived induced pluripotent stem cells (Kilpinen et al., 2017, E-MTAB-4057). The top 250 up- and 250 downregulated genes were selected to calculate stem cell identity and fibroblast identity for each cell as the sum of the number of respective genes expressed per cell and the cumulative expression signal of all genes as follows:

identity=n+∑logTPM

The identity is the stem cell or fibroblast identity, n the number of genes expressed and Σ⁡log TPM is the cumulative gene expression strength. Finally, stem cell identity and fibroblast identity for each cell were combined into a final stemness score as follows:

$$\mathrm{stemness}\;\mathrm{score}=\frac{\mathrm{stem}\;\mathrm{cell}\;\mathrm{identity}+1}{\mathrm{fibroblast}\;\mathrm{identity}+1}$$

### RNA Fluorescence *in Situ* Hybridization

RNA-FISH was carried out using RNAscope Multiplex Fluorescent Reagent Kit v2 and probe Hs-DPEP1-C2 combined with Hs-PDGFRA-C3 (ACD-bio, Newark, California) according to manufacturer’s protocol.

### Culture of Human Primary Skin Cells

Keratinocytes were cultured in KGM-Gold keratinocytes growth medium (Lonza, Basel, Switzerland) and fibroblasts in DMEM containing 10% (v/v) fetal bovine serum (FBS) (Biowest, Nuaillé, France) and 1% (v/v) penicillin/streptomycin (PS) (Thermo Fisher Scientific, Waltham, Massachusetts) at 37°C, 5% CO_2_ and 90% humidity. Treatment of skin cells with secreted proteins Activin A, MDK (both Merck, Darmstadt, Germany) and RBP4 (Abcam, Cambridge, United Kingdom) was done for 72 h.

### 3D Skin Equivalents and Histology

3D skin equivalents were generated as previously described (Boehnke et al., 2007). Hyalograft-3D was replaced with M3-II Bemcot scaffold (Asahi Kasei, Tokyo, Japan). During the last 6 (out of 12) weeks of culture, 100 ng/ml Activin A was added to the medium. Sections of 3D skin equivalents were stained with Diff-Quick (Labor + Technik Eberhard Lehmann, Berlin, Germany) according to manufacturer’s protocol. Epidermal thickness was determined using Fiji software (Schindelin et al., 2012).

### siRNA Knockdown

*COL11A1*, *DPEP1* and *MYL4* were selected as genes most upregulated in subpopulation 4a (Supplementary Table S2), especially young subpopulation 4a (Supplementary Table S3), while *HES1*, *CTNNB1*, *SOX2*, and *SOX11*, were selected as genes accounting for “stemness” GO-terms. For the knockdown, fibroblasts were incubated with 10 nM siRNAs (Supplementary Table S7), Lipofectamine^®^ RNAiMAX and Opti-MEM Reduced-Serum Medium (Thermo Fisher Scientific Waltham, Massachusetts) for 24 h, according to manufacturer’s protocol. After another 48 h, differentiation potential and/or proliferation were assessed.

### Analysis of Differentiation Potential

Fibroblasts were cultured in adipogenic differentiation medium (Lonza, Basel, Switzerland) for 2 weeks and stained with 1:1,000 HCS LipidTOX™ Deep Red Neutral Lipid Stain (Thermo Fisher Scientific, Waltham, Massachusetts). Images were taken using a Scan R high content screening station (Olympus, Tokyo, Japan). The percentage of differentiated fibroblasts was quantified using Scan R analysis software version 3.1.1. Fibroblasts were cultured in chondrogenic differentiation medium (Promocell, Heidelberg, Germany) for 2 to 3 weeks and time in days was measured until three-dimensional cartilage-like structure formed (Supplementary Figure S7).

### Proliferation assay and Determination of Procollagen Type I c-Peptide Concentration

BrdU cell proliferation ELISA (Roche, Basel, Switzerland) and procollagen type I c-peptide ELISA (Takara, Kyoto, Japan) were performed according to the manufacturer’s protocols and analyzed on a Tecan infinite M200 microplate reader (Tecan, Männedorf, Switzerland).

## Results

**FIGURE 1:**
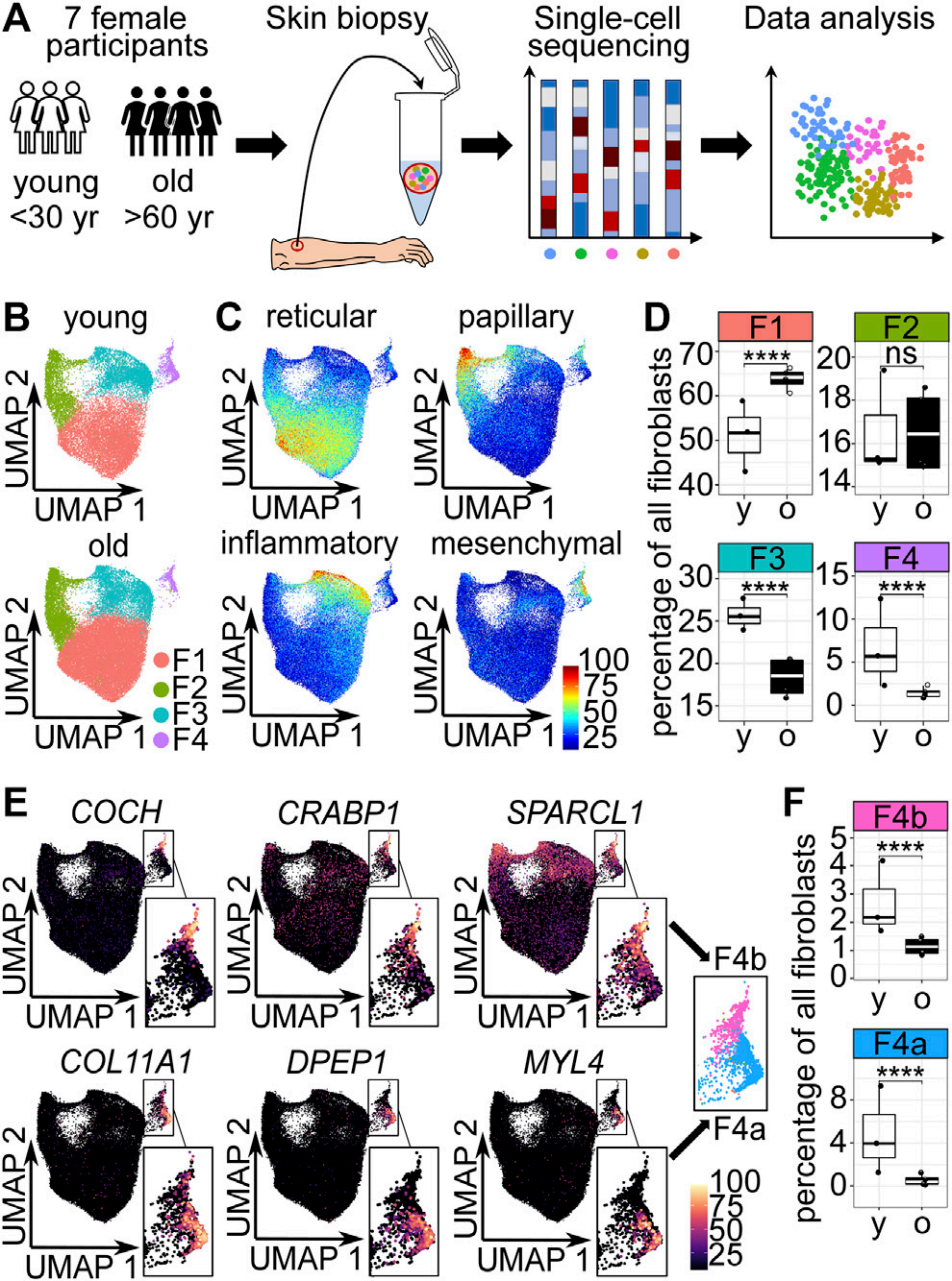
The cellular composition of dermal fibroblasts changes upon aging. **(A)** Experimental design. **(B)** Uniform Manifold Approximation and Projection (UMAP) plots of fibroblasts of young and old participants. **(C)** Expression of reticular (*WISP2*, *SLPI*, *CTHRC1*, *MFAP5*, *TSPAN8*), papillary (*APCDD1*, *ID1*, *WIF1*, *COL18A1*, *PTGDS*), pro-inflammatory (*CCL19*, *APOE*, *CXCL2, CXCL3, EFEMP1*) and mesenchymal (*ASPN, POSTN, GPC3, TNN, SFRP1*) markers (Solé-Boldo et al., 2020) among fibroblasts in UMAP plots. **(D)** Box plot of percentages of cells in fibroblast populations young vs. old, **** *p*<0.0001 (Fisher’s exact test). **(E)** Marker expression in UMAP plots, subclustering of population 4 into 4a and 4b. **(F)** Box plot of percentages of cells in fibroblast populations 4a and 4b young, vs. old, **** *p*<0.0001 (Fisher's exact test). F1-F4b Fibroblast populations 1–4b, yr years, y young, o old.

To identify cell populations that are related to skin aging, we carried out scRNA-seq of whole-skin samples from 4 old (>60 years, mean age 74.25 ± 5.252) and 3 young (<30 years, mean age 25.33 ± 3.512) human female participants (Figure 1A, Supplementary Table S1). Importantly, all samples were from the same gender and body area to limit confounding effects. Evaluation of quality control metrics (Supplementary Figure S1) and subsequent filtering resulted in 32,283 young and 39,765 old cells. Data analysis of the cells from all seven participants resulted in 14 clusters with distinct expression profiles (Supplementary Figure S2A). All identified clusters contained cells from all participants (Supplementary Figure S2B). Comparison with known markers revealed that 14 clusters represent 11 known cell types of the skin (Supplementary Figure S2C). Fibroblasts were identified based on *COL1A2*, *DCN*, *LUM*, *PDGFRA* and *VIM* expression as previously described (Solé-Boldo et al., 2020). In total, 19,139 fibroblasts originated from the young participants and 31,010 fibroblasts from the old (Supplementary Figures S2C, D).

### Fibroblast Composition Changes Upon Aging

Unsupervised clustering identified 4 dermal fibroblast populations (Figure 1B), which correspond to the described reticular, papillary, pro-inflammatory and mesenchymal fibroblast populations by Solé-Boldo et al., 2020 (Figure 1C). To identify potentially age-relevant populations, we analyzed cell numbers of fibroblast populations of young and old participants. The comparison revealed that the odds were significantly 1.685-fold greater that a fibroblast from the old participants was a fibroblast of the reticular population (population 1) than a fibroblast from the young participants (OR 1.685, *p* < 0.0001, 95% CI 1.624 to 1.748, Fisher’s exact test). The odds that a fibroblast belonged to the papillary population (population 2) was not significantly altered in young versus old participants. Compared to the old participants, the fibroblasts from the young participants were more likely to be a pro-inflammatory fibroblast (population 3) and mesenchymal fibroblast (population 4), respectively (pro-inflammatory population: OR 1.666, *p* < 0.0001, 95% CI 0.7971 to 1.741, mesenchymal population OR 4.070, *p* < 0.0001, 95% CI 1.835 to 5.078, Fisher’s exact test) (Figures 1B–D). However, with a 4-fold decrease, the strongest relative change in cell number was observed in the mesenchymal population upon aging (Figure 1D). Interestingly, visual inspection of Uniform Manifold Approximation and Projection (UMAP) plots displaying fibroblasts of young and old participants revealed that especially the lower part of the mesenchymal population was lost upon aging (Figure 1B). Analysis of marker gene expression revealed that mesenchymal population 4 could be subclustered into two distinct parts, subpopulation 4a and 4b (Figure 1E). The odds that a fibroblast from the old participants belonged to subpopulation 4a was about 7-fold decreased, compared to a fibroblast from the young participants (OR 7.097, *p* < 0.0001, 95% CI 6.051 to 8.350, Fisher’s exact test). The odds that a fibroblast of the old participants belonged to subpopulation 4b was approx. 2-fold decreased, compared to a fibroblast from the young participants (OR 2.258, *p* < 0.0001, 95% CI 1.961 to 2.602, Fisher’s exact test) (Figure 1F). Consequently, the cell number analysis confirmed that the subpopulation 4a was specifically decreased upon aging.

In summary, this suggests that the mesenchymal population 4, especially subpopulation 4a, could be age-relevant.

### Subpopulation 4a Represents the Dermal Sheath

**FIGURE 2:**
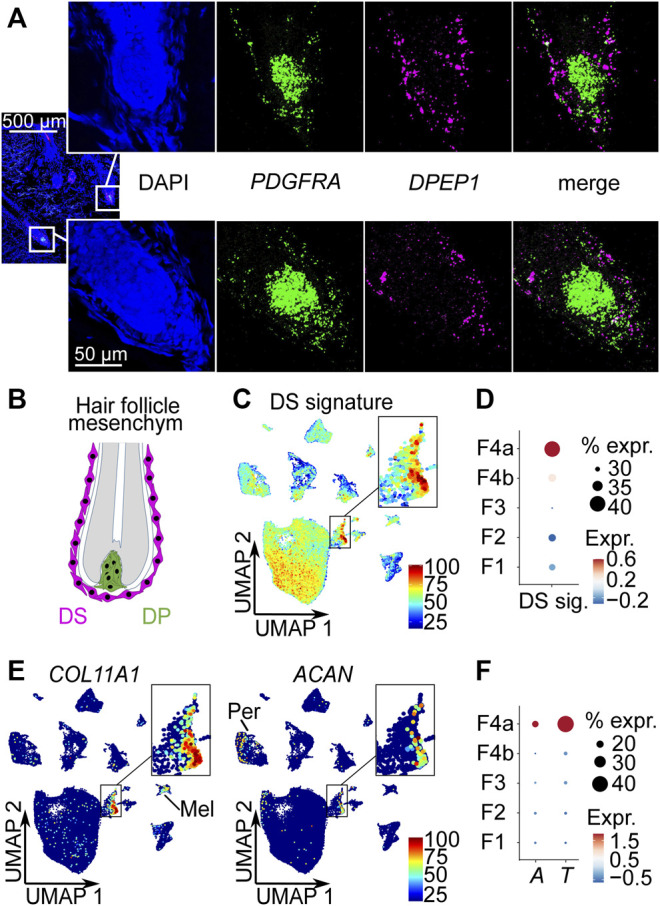
Subpopulation 4a represents the dermal sheath (DS). **(A)** RNA-FISH co-staining of subpopulation 4a marker *DPEP1* with *PDGFRA*, n = 5. **(B)** Mesoderm-derived cell types of the hair follicle: DS cells, dermal papilla (DP) cells. Average expression of top 10 DS signature genes (*CCDC80*, *COL11A1*, *TNMD*, *LRRC15*, *COL12A1*, *FGL2*, *CD200*, *FAM101B*, *SPARC*, *IGFBP7*) (Shin et al., 2020) **(C)** among all cell types in Uniform Manifold Approximation and Projection (UMAP) plot and **(D)** among fibroblast populations in dot plot. **(E)** Expression of DS-specific genes *COL11A1* and *ACAN* among all cell types in UMAP plots. **(F)** Dot plot of the expression of DS marker genes *ACTA2* (A) and *TAGLN* (T) among fibroblast populations. % expr. Percent expressed, expr. average expression, Mel melanocytes, Per pericytes.

scRNA-seq data analysis predicted *DPEP1* as one of the most significantly upregulated genes in the young subpopulation 4a (Supplementary Tables S2, S3). In all other fibroblast populations, the fraction of fibroblasts expressing *DPEP1* was less than 1% (Supplementary Table S2). To experimentally validate subpopulation 4a, we performed *DPEP1* RNA Fluorescence *In Situ* Hybridization. We also included *PDGFRA* as a pan-fibroblast marker. The results showed that *DPEP1* expression was localized in the mesoderm-derived dermal sheath (DS) surrounding the concentric layers of epidermal cells of the hair shaft and root sheath. More specifically, *DPEP1* expression was found in the DS of the hair bulb including the dermal sheath cup (DSC) (Figures 2A,B).

To further confirm subpopulation 4a as DS cells, we analyzed a published mouse DS gene signature (Shin et al., 2020), the expression of DS specific markers *COL11A1* and *ACAN*, and the expression of the smooth muscle-related genes *ACTA2* (Urabe et al., 1992; Hagner et al., 2020) and *TAGLN* (Heitman et al., 2020) in our scRNA-seq dataset. The analysis of the top 10 most upregulated mouse DS signature genes (*CCDC80*, *COL11A1*, *TNMD*, *LRRC15*, *COL12A1*, *FGL2*, *CD200*, *FAM101B*, *SPARC*, *IGFBP7*) clearly marked subpopulation 4a as DS cells (Figures 2C,D) among all cell types (Figure 2C) as well as among the fibroblasts (Figure 2D). Among these genes, especially *COL11A1*, *TNMD* and *SPARC* were strongly upregulated in our DS population (Supplementary Figure S3A). Additionally, *POSTN*, *PMEPA1* and *DPEP1*, which are specifically upregulated in our DS population were also among the total of 36 upregulated genes of the mouse DS population by Shin et al. (2020) (Supplementary Figure S3B). Furthermore, we found that the DS-specific gene *COL11A1* (Shin et al., 2020) was strongly and rather specifically expressed in our DS population (Figure 2E), while being the most significantly upregulated marker of our young DS population (Supplementary Table S2). Melanocytes also showed some *COL11A1* expression, but the expression level was substantially lower compared to the DS population (Figure 2E). As shown before (Shin et al., 2020), *ACAN* was not expressed by all DS cells and also at lower levels compared to *COL11A1* (Figure 2E), with pericytes being the only other population expressing *ACAN*. Among all the cell types in our dataset, the DS population was the only population that expressed both specific mouse DS markers. Finally, expression of well-known DS smooth muscle-related genes *ACTA2* (Urabe et al., 1992; Hagner et al., 2020) and *TAGLN* (Heitman et al., 2020) were significantly (*p* < 0.0001, FindAllMarkers function with implemented Wilcoxon Rank Sum test) enriched in our DS population (Figure 2F, Supplementary Figure S4A, Supplementary Table S2) among the fibroblasts populations, while *MYH11* and *MYL9* (Heitman et al., 2020) were not significantly enriched (Supplementary Figure S4A, Supplementary Table S2). In comparison to all cell types, pericytes and smooth muscle cells (including arrector pili muscle cells) expressed higher levels of smooth muscle-related genes *ACTA2*, *TAGLN*, *MYH11* and *MYL9* (Supplementary Figure S4B). Additionally, we found other muscle-related genes (*MEF2C*, *MYL4* and *PPP1R14A*) expressed (Supplementary Figure S4C) among the top ten upregulated genes of our young DS population (Supplementary Table S2). Moreover, the receptor LGR4 that was reported to mediate Wnt signalling in DS cells (Hagner et al., 2020), was significantly enriched in the young DS population (*p* < 0.0001, FindAllMarkers function with implemented Wilcoxon Rank Sum test) (Supplementary Figure S5, Supplementary Table S2). Taken together, the enrichment of DS signature genes, especially the DS specific genes *COL11A1* and *ACAN* and the upregulation of muscle-related genes, including the previously reported *ACTA2* and *TAGLN* as well as unreported ones e.g., *MYL4*, strongly suggest that the identified population represents the DS.

### Validation of Dermal Sheath Population and its Loss Upon Aging in Independent scRNA-seq Datasets

**FIGURE 3:**
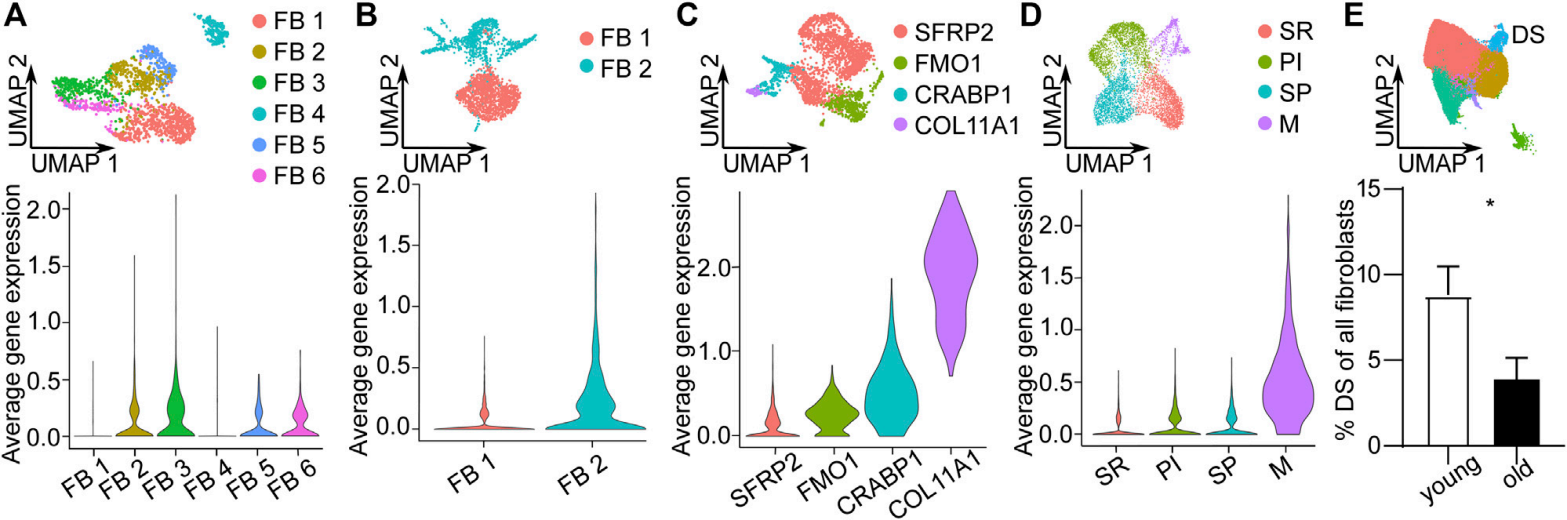
Validation of Dermal sheath (DS) population and its loss upon aging in independent scRNA-seq datasets. Uniform Manifold Approximation and Projection (UMAP) plots of fibroblast populations from **(A)** Vorstandlechner et al., 2020, **(B)** Rojahn et al., 2020, **(C)** Tabib et al., 2020 and **(D)** Solé-Boldo et al., 2020. Violin plots of the average expression of the ten most upregulated genes of DS population (*COL11A1*, *DPEP1*, *POSTN*, *TAGLN*, *MEF2C*, *MYL4*, *TNMD*, *WFDC1*, *GPC3*, *PPP1R14A*) in these datasets. **(E)** UMAP plot of fibroblasts of other datasets integrated together with our dataset. Percentage of DS population young vs. old, n = 8 (young), n = 9 (old). *p < 0.05 (two-tailed unpaired *t*-test), mean +/- SEM. FB Fibroblasts, SR secretory-reticular, PI pro-inflammatory, SP secretory-papillary, M mesenchymal.

To validate the DS population and its loss upon aging in independent datasets, we analyzed published scRNA-seq datasets from human skin (Tabib et al., 2018; Rojahn et al., 2020; Solé-Boldo et al., 2020; Vorstandlechner et al., 2020). We could identify cells expressing the top 10 DS marker genes in all 4 datasets (Figures 3A–D). Interestingly, DS cells were clustered in one of the minor, potentially pluripotent population expressing *COL11A1*, *DPEP1* and *RBP4* in one dataset (Figure 3C). Further integration of all datasets also confirmed that the number of DS cells was significantly (*p* < 0.01, unpaired *t*-test) decreased upon aging (Figure 3E). These findings confirm DS cells in independent datasets and further validate their loss upon aging.

### The Dermal Sheath Population Likely Contains Progenitor Cells that are Lost Upon Aging

**FIGURE 4:**
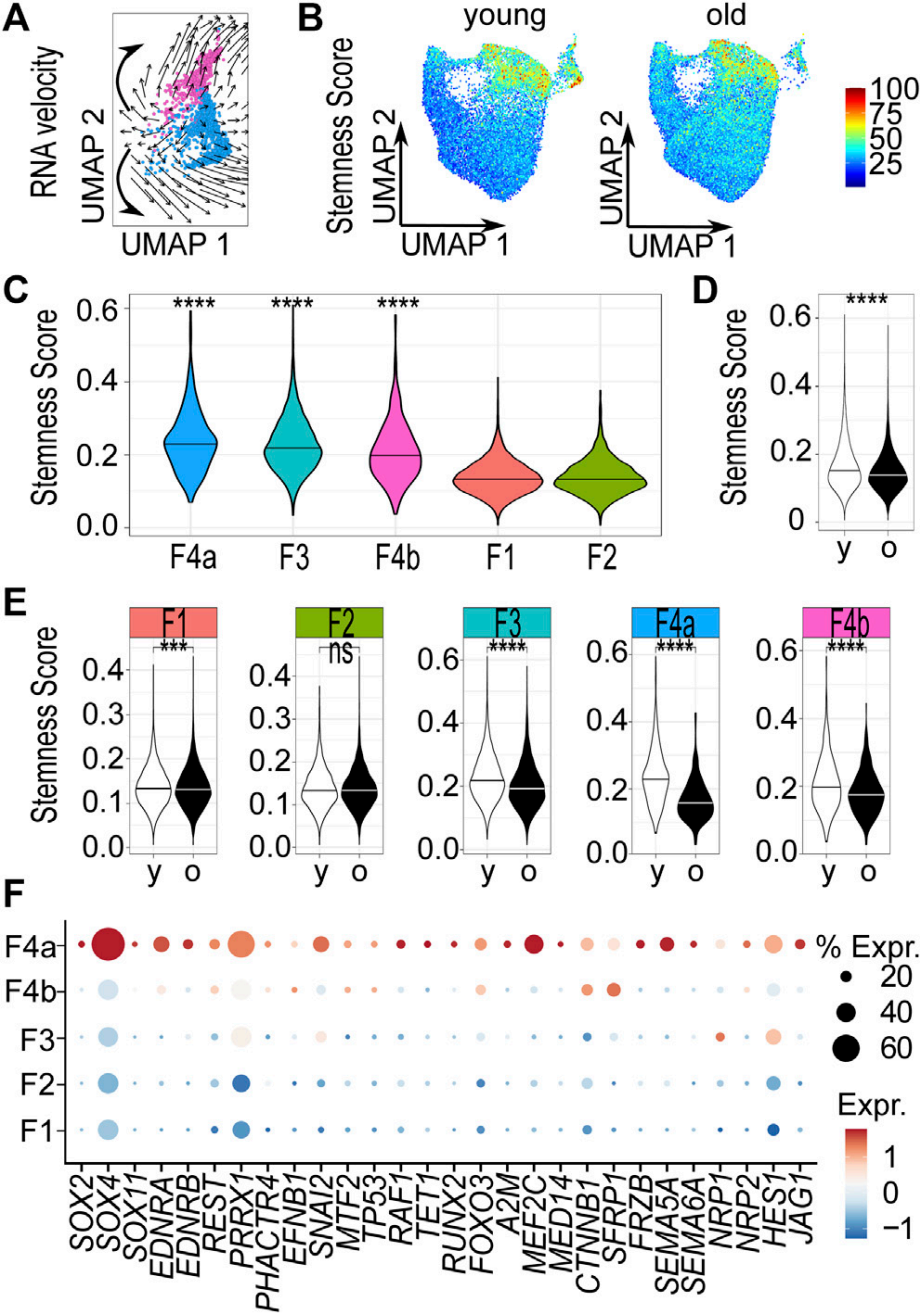
The dermal sheath population likely contains progenitor cells that are lost upon aging. **(A)** RNA velocity analysis of mesenchymal population 4, computed arrows indicating the extrapolated future states of the cells, bold hand-drawn arrows indicating 2 cell fates. **(B)** Stemness score of each cell in Uniform Manifold Approximation and Projection (UMAP) plots young vs. old. Violin plots of stemness score of **(C)** young populations, **(D)** young vs. old fibroblasts and **(E)** young vs. old populations. ***p < 0.001, ****p < 0.0001 (unpaired Wilcoxon rank sum tests). **(F)** Dot plot of the expression of genes accounting for “stemness” gene ontology terms by fibroblast populations. F1-4b Fibroblast populations 1–4b, y young, o old. % expr. Percent expressed, expr. average expression.

As the DSC is known to include hair follicle dermal stem cells, we tested whether we could find indications for stem cell characteristics of the DS population. To this end, we applied three different approaches: In the first approach, we applied RNA velocity to predict differentiation trajectories. The analysis revealed a potential origin of differentiation within the mesenchymal population. The computed arrows (the extrapolated future states of the cells) pointed in the direction of the upper part of the mesenchymal population, which expresses high levels of dermal papilla specific markers (Figure 1E) and the lower part of the mesenchymal population. These findings are consistent with the notion that the progenitors acquire the 2 cell fates described by Shin et al. (2020) in mice (Figure 4A).

In the second approach, we compared our scRNA-seq data with expression data of stem cells derived from dermal fibroblasts (Kilpinen et al., 2017). We therefore defined a stemness score, comprising the 500 most differentially expressed (250 up- and 250 downregulated) genes between stem cells and fibroblasts (see Materials and Methods for details). Uniform Manifold Approximation and Projection revealed distinct age- and population-dependent differences in the stemness score of fibroblasts (Figure 4B). Among the young samples, the DS population had the highest (*p* < 0.0001, one-sided unpaired Wilcoxon rank sum test) score (Figure 4C). We also noticed that the stemness score of young fibroblasts was significantly (*p* < 0.0001, two-sided unpaired Wilcoxon rank sum test) increased compared to old fibroblasts, suggesting that young skin fibroblasts are more closely related to stem cells than old skin fibroblasts (Figure 4D). Furthermore, a strong and significant (*p* < 0.0001, two-sided unpaired Wilcoxon rank sum test) reduction of the stemness score was specifically observed in the DS population (Figure 4E) upon aging.

In a third approach, we carried out Gene Ontology (GO) term analyses with representative genes of all young and old fibroblasts populations, respectively (Supplementary Table S2) to support our hypothesis that we have identified the DS population, including the progenitor cells with stem cell characteristics. Therefore, we specifically searched for GO-terms related to stemness among all populations. Our results showed that genes accounting for the GO terms “neuronal stem cell population maintenance”, “stem cell population maintenance”, “stem cell differentiation” and “stem cell development” (“stemness” GO terms) were exclusively enriched among representative genes of the young DS population. In contrast, genes accounting for “stemness” GO terms were not found to be enriched in the old DS population. Apart from genes accounting for “regulation of hematopoietic stem cell differentiation” in papillary population 2, no GO terms related to stemness were found in any of the other fibroblast populations (Supplementary Table S4). Similarly, expression of genes accounting for “stemness” GO terms of DS population was specifically increased in the DS population (Figure 4F). Taken together, these findings suggest that the DS population contains progenitor cells. Furthermore, our analyses also support an age-related loss of the DS population and its stem cell features.

### Characteristic Genes of the Dermal Sheath Regulate Stem Cell Characteristics

**FIGURE 5:**
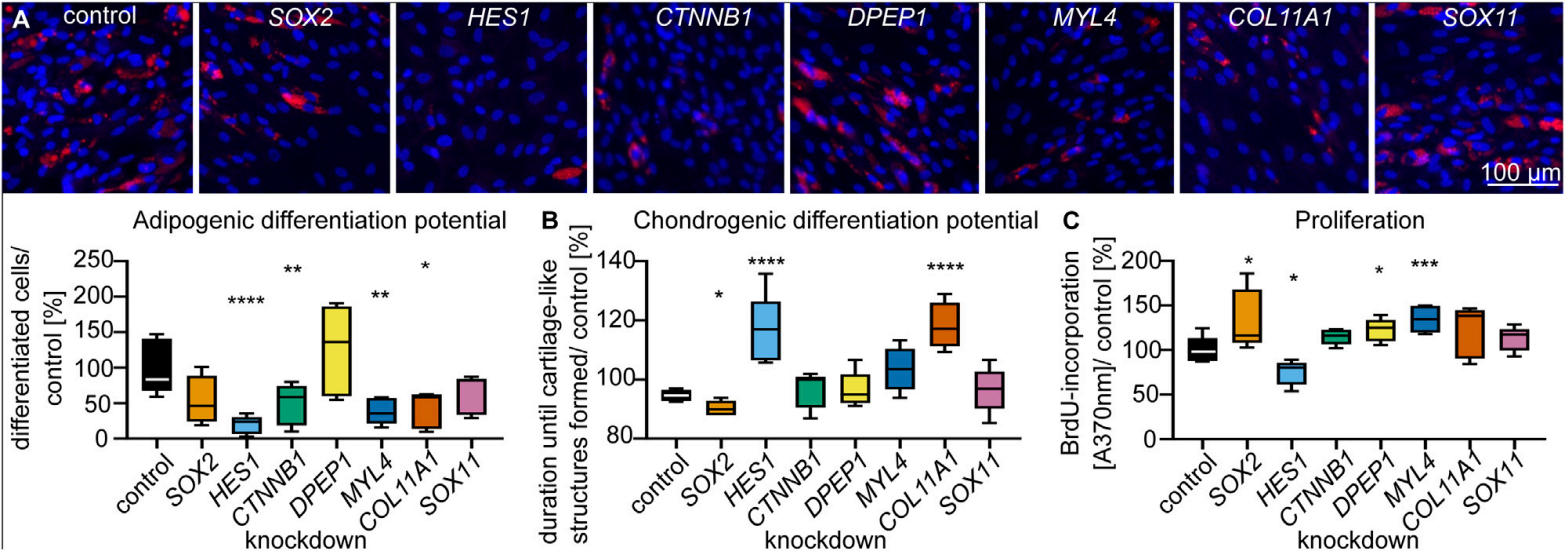
Characteristic genes of the dermal sheath regulate stem cell characteristics. **(A)** Adipogenic differentiation potential, **(B)** chondrogenic differentiation potential and **(C)** proliferation, 72 h after knockdown of DS genes. n = 5, *p < 0.05, **p < 0.01, ***p < 0.001, ****p < 0.0001 (RM one-way ANOVA with Dunnett multiple testing correction).

To functionally characterize DS-related genes in a model of basic mesenchyme-derived cells, we carried out siRNA-mediated knockdown experiments in primary dermal fibroblasts from young (age <30 years) donors. Based on their expression characteristics (see Materials and Methods for details, Figure 4F, Supplementary Tables S2, S3), we selected *COL11A1*, *DPEP1*, *MYL4*, *HES1*, *CTNNB1*, *SOX2* and *SOX11* as candidate genes and verified their expression by qPCR (Supplementary Table S5). Knockdown efficiency was on average > 75% (Supplementary Figure S6). As there are no available human *in vitro* models to mimic the differentiation of mesenchymal progenitors into dermal papilla cells/ dermal sheath cells, we tested the differentiation potential for other differentiation trajectories of mesenchymal progenitors (adipose and cartilage, (Dominici et al., 2006)). Furthermore, we tested proliferation capacity as a requirement for self-renewal (Rich, 2015). The results showed that the proportion of fibroblasts that accumulated triglycerides in lipid droplets was significantly (*p* < 0.05, RM one-way ANOVA, Dunnett multiple testing correction) decreased after *HES1*-, *CTNNB1*-, *MYL4*- and *COL11A1*-knockdown compared to controls (Figure 5A). Also, the time until cartilage-like three-dimensional structures formed (Supplementary Figure S7), was significantly (*p* < 0.05, RM one-way ANOVA, Dunnett multiple testing correction) prolonged after *HES1*- and *COL11A1*-knockdown compared to controls (Figure 5B). Both results indicate a functional role of DS-related genes in the differentiation capacity into different lineages. Furthermore, *HES1*-knockdown fibroblasts also showed decreased proliferation, indicating that this gene may affect the self-renewal of stem cells (Figure 5C).

### Secreted Proteins of the Dermal Sheath Mediate Paracrine Effects on Fibroblasts and Keratinocytes and Contribute to a Juvenile Skin Phenotype

**FIGURE 6:**
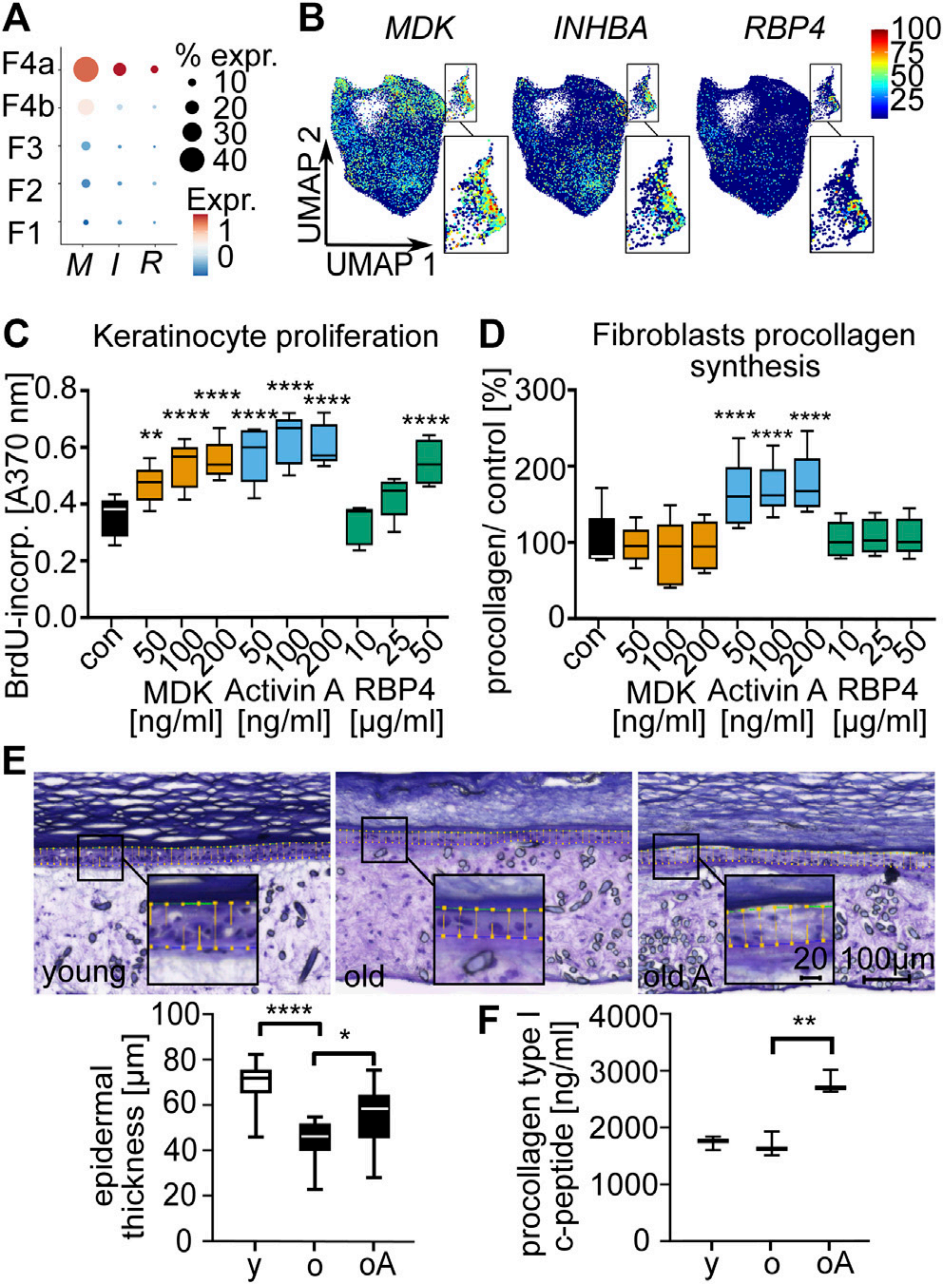
Secreted proteins of the dermal sheath mediate paracrine effects on fibroblasts and keratinocytes and contribute to a juvenile skin phenotype. Expression of *INHBA* (I), *MDK* (M) *and RBP4* (R) among the fibroblast populations **(A)** in dot plot and **(B)** in Uniform Manifold Approximation and Projection plots. Box plots of **(C)** proliferation of old keratinocytes and **(D)** procollagen type I c-peptide synthesis of old fibroblasts after treatment with secreted proteins MDK, Activin A and RBP4 for 72 h, n = 5, *p < 0.05, **p < 0.01, ***p < 0.001, ****p < 0.0001 (RM one-way ANOVA with Dunnett multiple testing correction). **(E)** Epidermal thickness of 3D skin equivalents stained with Diff-Quick (microscopy images) and **(F)** procollagen synthesis. Comparison of young (y) vs. old (o) and old vs. old Activin A treated (100 ng/ml for 6 weeks, old A/oA) (box plots), n = 3, 5 slices were used for each n to determine epidermal thickness, *p < 0.05, **p < 0.01, ***p < 0.001, ****p < 0.0001 (two-tailed un/paired *t*-test).

Finally, we also analyzed the paracrine effects of the DS by secreted proteins. To this end, we treated primary keratinocytes and fibroblasts with DS-secreted proteins. Based on expression characteristics and protein availability, we selected MDK, Activin A (a dimer of two INHBA subunits) and RBP4 as candidate proteins (Supplementary Table S6, Figures 6A,B). Keratinocyte proliferation was significantly (*p* < 0.001, RM one-way ANOVA, Dunnett multiple testing correction) increased after treatment with all tested proteins (Figure 6C). We also observed that fibroblast procollagen type I c-peptide production was significantly (*p* < 0.0001, RM one-way ANOVA, Dunnett multiple testing correction) increased after treatment with Activin A (Figure 6D). To confirm these effects in a 3D skin model, we treated human skin equivalents with Activin A. The protein significantly (*p* < 0.05, paired *t*-test) increased epidermal thickness (Figure 6E) and also specifically increased (*p* < 0.01, paired *t*-test) procollagen type I c-peptide production in 3D skin equivalents consisting of fibroblasts from old donors (Figure 6F). Our results thus suggest that Activin A is a mediator of beneficial paracrine effects of the DS on both major cellular components of the skin, keratinocytes and fibroblasts, thereby contributing to a juvenile skin phenotype.

## Discussion

Very little is known about human dermal sheath cells, the pathways contributing to their differentiation capacity and their effects on skin homeostasis. We have used scRNA-seq to analyze transcriptomes of 72,048 cells from healthy human skin samples, including 50,149 fibroblasts. We provide the first characterization of human DS cells at the single-cell level, and describe their loss upon aging, their stem cell characteristics and contribution to a juvenile skin phenotype.

Our results show age-related changes in fibroblast populations. Among the populations that were affected by aging, subpopulation 4a was of particular interest, because it showed the strongest (about 7-fold) decline in cell number. The observed age-dependent reduction of subpopulation 4a may indicate an age-related reduction in DS cell numbers, consistent with recently published observations (Williams et al., 2021) describing a miniaturization of female hair follicles upon aging. Our findings thus provide an important extension of previous observations that described age-related changes across fibroblast populations, including a loss of fibroblast priming, skin aging-associated secreted proteins expression profiles upon aging (Solé-Boldo et al., 2020) and a geroprotective effect of *HES1* (Zou et al., 2021).

We also show that subpopulation 4a localized to the human DS compartment, demonstrated an enriched expression of mouse DS signature genes and the DS-specific genes *COL11A1* and *ACAN* (Shin et al., 2020). To facilitate DS contraction required for follicle regression, the mouse DS expresses the molecular machinery of smooth muscle cells (Heitman et al., 2020). Moreover, immunofluorescence of smooth muscle proteins (ACTA2, TAGLN, MYH11, MYL9) in scalp follicles suggested that their expression is also enriched in the human DS (Heitman et al., 2020). While the highest expression of smooth muscle-related genes was found in pericytes and smooth muscle cells including arrector pili muscle cells, *ACTA2* and *TAGLN* were upregulated in the DS population in comparison to other fibroblast populations. This finding is comparable to published scRNA-seq data in mice (Shin et al., 2020). Together, the localization in the DS, the enriched expression of mouse DS signature genes, the expression of DS-specific genes and comparable expression of muscle-related genes to mouse DS scRNA-seq data by Shin et al., 2020 suggest that we have identified the human DS population.

The DS is known to harbor hair follicle dermal stem cells exhibiting self-renewal and functioning as bipotent progenitors in mice (Rahmani et al., 2014; Hagner et al., 2020). Based on three independent approaches, including RNA velocity, the stemness score and GO terms, our findings indicate that the DS population includes progenitors and that their stem cell characteristics are lost upon aging in line with age-dependent decline in stem cell function previously reported (Oh et al., 2014).

Our findings also suggest that the DS-related genes *HES1*, *COL11A1*, *MYL4* and *CTNNB1* play a role in regulating stem cell characteristics. *HES1* was among the key genes accounting for “stemness” GO terms. Knockdown of the transcription factor in dermal fibroblasts significantly decreased adipogenic and chondrogenic differentiation capacity and reduced proliferation capacity, a fundamental property required for self-renewal (Rich, 2015). In addition, *HES1* has been described to be downregulated in dermal fibroblasts upon UV radiation, a known mechanism of extrinsic skin aging (Zou et al., 2021). Finally, dermal fibroblasts over-expressing *HES1* exhibited geroprotective effects including increased proliferation and attenuated cellular senescence (Zou et al., 2021). Our results indicate that geroprotective effects of *HES1* might be mediated by a role of *HES1* in stem cell features of the human DS.

*CTNNB1*, encoding an intracellular transducer of WNT signaling, was also among the key genes accounting for “stemness” GO terms, was significantly upregulated in the DS and fibroblasts showed a decreased adipogenic differentiation capacity upon its knockdown. WNT signaling plays an important role in the crosstalk of the DS and the DP. Mouse DP cells secrete R-spondins which increase hair follicle stem cell proliferation potentially via LGR6 (Hagner et al., 2020). Our results show that *LGR4* is significantly upregulated in the young DS (Supplementary Figure S5, Supplementary Table S2), which has been suggested to mediate the effects of R-spondins in cultured adult human colony-forming dermal progenitors (Hagner et al., 2020). Our findings thus indicate that WNT signalling also plays a role in the human DS by regulating differentiation capacity and that its activation is potentially induced by R-spondin binding to LGR4.

*COL11A1* was most significantly upregulated in the young DS cells and was expressed in less than 1% of fibroblasts of other populations. Knockdown of *COL11A1* decreased adipogenic and chondrogenic differentiation capacity. This is in line with the reported upregulation of *COL11A1* in human dermal fibroblasts that were cultured with demineralized bone powder to induce chondrocyte features (Yates and Glowacki, 2003). *Col11a1* expression is also highly increased in the neonatal mouse dermis and in mouse dermis that has been reprogrammed to a neonatal stage by epidermal beta catenin activation (Collins et al., 2011). Furthermore, *COL11A1* is upregulated upon injection of hyaluronic acid-based dermal fillers (Huth et al., 2020) and downregulation of *Col11a1* is associated with delayed wound healing (Gu et al., 2020). These observations might be attributed to our finding that *COL11A1* increases the differentiation capacity. Finally, our results also identified *MYL4* among the most significantly upregulated genes of the DS and confirm a functional role in adipogenic differentiation, thus identifying a new DS marker.

We further show that proteins secreted by the DS, especially Activin A, mediated rejuvenating effects on keratinocytes and dermal fibroblasts. Activin A is a homodimer consisting of two INHBA subunits. *INHBA* expression was significantly increased in the young DS. Other subunits of Activins (*IHNBB*, *IHNBC*, *INHBE*) and Activin antagonists (*FST, FSTL3* and *INHA*) were not enriched in the DS (Supplementary Table S2). Activin A increased keratinocytes proliferation and fibroblasts procollagen type 1 c-peptide production. Furthermore, epidermal thickness and procollagen type 1 c-peptide production were increased in 3D skin equivalents from old donors, thereby shifting an old skin phenotype towards a juvenile phenotype. In line with these results, mice lacking the Activin antagonist Fst have been shown to exhibit enhanced keratinocyte proliferation (Antsiferova et al., 2009). Furthermore, collagen synthesis is stimulated in mice overexpressing Activin A in keratinocytes (Wietecha et al., 2020). Further research will be required to explore transport mechanisms of secreted proteins within the skin, e.g. via exosomes, and the potential application of Activin A in anti-aging interventions.

## Data Availability

The generated scRNA-seq datasets for this study will be found in the Sequence Read Archive (SRA) https://www.ncbi.nlm.nih.gov/sra/PRJNA754272. Any other data are available from the corresponding authors upon reasonable request.
