# Supplementary material for: Single-Cell RNA Profiling of Human Skin Reveals Age-Related Loss of Dermal Sheath Cells and Their Contribution to a Juvenile Phenotype
Source: Front Genet. 2022 Jan 7;12:797747. doi: 10.3389/fgene.2021.797747 (PMC8776708; doi:10.3389/fgene.2021.797747)
Supplement: Supplementary file 3 [file DataSheet1.docx]

Supplementary Material

# Supplementary Materials & Methods

## Study participants

This single-cell RNA sequencing study was performed in accordance with the Declaration of Helsinki and approved by the Ethics Committee of the Medical Association of Hamburg (PV6054).

Three young (aged <30) and four old (aged >60) healthy female Caucasian participants were selected according to defined exclusion criteria (Table S8).

For validation and functional experiments skin from healthy, female Caucasian donors was purchased from Alphenyx, Marseille, France. A detailed overview of the participants/donors and experiments, skin biopsies and cells is given in Table S1.

## Droplet based scRNA-seq & data analysis

4 mm whole skin punch biopsies were obtained from the outer forearm of the seven participants by bioskin GmbH (Hamburg, Germany). Droplet based scRNA-seq was done as previously described (Sole-Boldo et al., 2020).

### Clustering and Cell Type Annotation

Data of each participant was pre-processed independently as follows: Raw reads were quality filtered and aligned to the human genome version GRCh37 by 10x Genomics Cell Ranger 2.1.0, (10x Genomics, Pleasanton, California) (Zheng et al., 2017). The feature-barcode matrices generated by Cell Ranger were used as input for the Seurat package, version 3.6.4 (Stuart et al., 2019), in R, version 4.0.5 (R Core Team, 2020, https://www.R-project.org/). Cells with less than 200 or more than 7,500 expressed genes or cells expressing more than 5% mitochondrial reads were removed. The raw counts were log-normalized (NormalizeData) and the 2,000 most variably expressed genes (FindVariableFeatures) were identified.

The pre-processed data was integrated according to Seurat standard integration protocol. Integration anchors were identified between our seven participants in the first 30 canonical correlation analysis (CCA) dimensions with default parameters (FindIntegrationAnchors). These anchors were used for the integration applying the default parameters and the first 30 CCA dimensions (IntegrateData). The integrated data were scaled (ScaleData) before performing a principle component analysis (PCA) of the first 100 dimensions (RunPCA). The dimensionality of the dataset was defined via manual inspection of the first 100 dimensions using the ElbowPlot (flattening in percentage of variance) and ScoreJackStraw (sharp drop-off in significance) functions. Based on these analyses the first 33 dimension were used to construct a Shared Nearest Neighbor (SNN) Graph (FindNeighbors) using the default parameters. Cells were clustered by applying a SNN modularity optimization-based clustering algorithm with a resolution of 0.2 (FindClusters). Cluster with an average of less than 10 cells per participant, were removed. For subsequent visualization of the dataset the Uniform Manifold Approximation and Projection (UMAP), a non-linear dimensional reduction technique, was performed with the first 33 dimensions (RunUMAP).

Clusters were assigned to different cell types by visual projection of the calculated average expression of a particular marker gene set per cell (FeaturePlot) as previously described (Sole-Boldo et al., 2020). As marker gene set for Schwann cells CRYAB (Lim et al., 2017), MP2 (Stettner et al., 2018), GPM6B (Bang et al., 2018), S100B (Mata et al., 1990), NRXN1 (Castro et al., 2020) and for smooth muscle cells ACTA2 (Yuan, 2015), ACTG2 (Halim et al., 2016), CD146 (Roostalu et al., 2018), DES (Paulin and Li, 2004) was used.

Clusters assigned to fibroblast populations were extracted from the Seurat object, which contained all cell types, by applying the subset function.

Using the analysis pipeline as described above, a resolution of 2.6 was used for the subclustering of population 4 (FindClusters). The newly generated cluster identities were used to re-assign the subpopulations of population 4 to 4a and 4b, whereas all other fibroblast populations kept the identities of the initial clustering (resolution 0.2).

### RNA velocity

To receive the LOOM-files the following steps were carried out: First, the current annotation of the human genome GRCh37 was downloaded from ensembl.org (Yates et al., 2020) in GTF-file format. Second, corresponding information of repeats on the human genome was downloaded from repeatmasker.org (Smit et al., 2013-2015) and the standard RepeatMasker output was converted into GFF3-file format using the RepeatMasker function rmOutToGFF3.pl, version 4.1.0 (Smit et al., 2013-2015). The obtained GFF3-file was converted into GTF-file format by the rtracklayer package, version 1.50.0 (Lawrence et al., 2009). Third, samtools, version 1.10 (Li, 2011, Li et al., 2009), was applied to the BAM-files generated by Cell Ranger to obtain the corresponding indexed BAI-files. Finally, the genome annotation files, feature-barcode matrix, the BAM-file and the indexed BAI-file for each participant separately were used to receive LOOM-files by velocyto, version 0.17.17 (La Manno et al., 2018).

LOOM-files of participants were merged into one single LOOM-file using the loompy.combine function of the loompy package, version 3.0.6 (https://github.com/linnarsson-lab/loompy), in python, version 3.6 (VanRossum and Drake, 2009). ://github.com/linnarsson-lab/loompy), in python, version 3.6 (VanRossum and Drake, 2009). Population 4 of the Seurat object, which was generated in the previous clustering step, was used for the RNA-velocity analysis. RNA velocity was performed on LOOM-files as follows using the velocyto.R package, version 0.6 (La Manno et al., 2018), in R, version 4.0.5: Genes were filtered based on the minimum average expression magnitude in at least one of the clusters from the corresponding Seurat object (0.2 as cut off for the spliced matrix, and 0.05 for the unspliced matrix; filter.genes.by.cluster.expression). The UMAP-embeddings were extracted from the Seurat object and converted into cell-cell distance via the armaCor function. The filtered spliced, unspliced matrices and the cell-cell distance were used to estimate RNA velocity by gene-relative slopes setting kCells to 20, deltaT to 1 and fit.quantile to 0.02 (gene.relative.velocity.estimates). The estimated RNA velocity was projected onto the UMAP applying the show.velocity.on.embedding.cor function using a neighborhood size of 300, a sqrt-scaling of the velocity, 40 grid points along each axis, an arrow scale of 5 and a minimal cell mass of 0.5 around each grid point.

### Functional characterization of fibroblast populations

Stemness scores for individual cells were calculated using marker genes derived from gene expression data generated by (Kilpinen et al., 2017, E-MTAB-4057). A stemness signature was generated by calculating differentially expressed genes between primary human dermal fibroblasts and stem cells from quantile normalized, log transformed intensities using the limma R package, utilizing a paired design matrix to account for inter-individual donor variation. The top 250 positive and 250 negative markers from the signature were selected to calculate stem cell identity and fibroblast identity for each cell as the sum of the number of respective marker genes expressed per cell and the cumulative expression signal of all marker genes as follows:

$$identity=n+\sum logTPM$$

with $identity$being the stem cell or fibroblast identity, $n$ the number of markers expressed and $\sum logTPM$ being the cumulative marker expression strength. Finally, stem cell identity and fibroblast identity for each cell were combined into a final stemness score as follows:

$$stemness score = \frac{stem cell identity+1}{fibroblast identity+1}$$

To identify genes with enriched expression in each fibroblast populations of the young and old participants and in young versus old subpopulation 4a fibroblasts, we used the FindAllMarkers function (p-value <0.05, min.pct. 0.05, log2FC>0.1), Seurat version 4.0.0 (Hao et al., 2020).

Gene ontology (GO) Enrichment Analysis (Ashburner et al., 2000, Gene Ontology, 2021, Mi et al., 2019) was done with representative genes (log2 fold change > 0.1) of each fibroblast population in the young and old participants (settings: biological process, Fisher's Exact test, False Discovery Rate correction, p-value <0.05).

### Validation of subpopulation 4a in independent scRNA-seq datasets of human skin

For validation of subpopulation 4a, scRNA-seq datasets by (Rojahn et al., 2020, GSE153760) and (Solé-Boldo et al., 2020, GSE130973) were used. Furthermore, datasets from Tabib et al. 2018 and Vorstandlechner et al. 2020 were kindly provided by the authors. All datasets were analysed according to the original publication using Seurat package, version 4.0.0 (Hao et al., 2020), in R, version 4.0.3 (R Core Team, 2020, https://www.R-project.org/). The average expression of subpopulation 4a’s ten most upregulated genes (COL11A1, DPEP1, POSTN, TAGLN, MEF2C, MYL4, TNMD, WFDC1, GPC3, PPP1R14A) was calculated for each fibroblast population in all datasets. These average expression values were then visualized using violin plots (VlnPlot).

### Validation of age-dependent loss of fibroblast subpopulation 4a

Other scRNA-seq datasets of human skin were integrated together with our dataset as described above. The first 30 dimensions and a resolution of 0.4 were used for clustering of the cells.

To identify cluster equivalent to subpopulation 4a within the newly generated Seurat object, the cell identifiers of subpopulation 4a fibroblasts from our dataset were used. One cluster contained most of the original cell identifiers and was subsequently further subdivided to precisely delimit subpopulation 4a. A resolution of 1.4 was used for the subclustering (FindClusters).

### Comparison of dermal sheath signature with gene expression of populations

36 connective tissue signature genes (Shin et al., 2020) were compared with the gene expression of populations using a modified DotPlot. The original DotPlot function implemented in Seurat, version 3.6.4, was modified to display the average scaled gene expression as well as the average percent expression for a given set of genes by one dot in the final plot. Modifications to the DotPlot function were introduced after the final data.plot matrix was generated, which contains the average expression (avg.exp), scaled average expression (avg.exp.scaled) and percent expression (pct.exp) for each feature per group. Based on a given list-object with named list elements containing various genes, the corresponding features were extracted per list-element from the data.plot matrix and the averages of avg.exp, avg.exp.scaled and pct.exp, respectively, were calculated for each group. Thus, a new data frame data.plot2 was obtained similar to the original data.plot matrix containing the average gene expression, percent expression, scaled gene expression, element list name and group-ids per gene set. Finally, the data were plotted with ggplot2, version 3.3.3 (Wickham, 2016, https://ggplot2.tidyverse.org).

## RNA Fluorescence in situ hybridization (RNA-FISH)

RNA-FISH was carried out on 16 µm human skin sections of five donors (Table S1) using RNAscope Multiplex Fluorescent Reagent Kit v2 and probes Hs-DPEP1-C2 combined with Hs-PDGFRA-C3 (ACD-bio, Newark, California) according to manufacturer’s protocol. Images were taken at Leica TCS SP5 II confocal imaging system (Leica Microsystems, Wetzlar, Germany) at 10x and 40x magnifications.

## Isolation and culture of human primary skin cells

The skin was incubated in Dulbecco's Phosphate-Buffered Solution (DPBS) (Thermo Fisher Scientific, Waltham, Massachusetts) containing 2.4 U/ml dispase II (Roche, Basel, Switzerland) at 4°C overnight and separated into epidermis and dermis by manual dissection. The keratinocytes were isolated from the epidermis by trypsinization with 0.25 % Trypsin/EDTA (Thermo Fisher Scientific, Waltham, Massachusetts) at 37°C for 10 min. The fibroblasts were isolated from the dermis by incubation in DMEM (Thermo Fisher Scientific, Waltham, Massachusetts) containing 4 mg/ml Collagenase D (Roche, Basel, Switzerland), 2 mg/ml Hyaluronidase (Merck, Darmstadt, Germany), 100 U/ml DNAse 1 (Thermo Fisher Scientific, Waltham, Massachusetts) at 37°C for 3 h.

Keratinocytes were cultured in KGM-Gold keratinocyte growth medium (KGM-Gold) (Lonza, Basel, Switzerland) and fibroblasts in DMEM containing 10 % (v/v) fetal bovine serum (FBS) (Biowest, Nuaillé, France) and 1 % (v/v) penicillin/streptomycin (PS) (Thermo Fisher Scientific, Waltham, Massachusetts) at 37°C, 5 % CO_2_ and 90 % humidity.

## Treatment with secreted proteins

Treatment with secreted proteins was done for 72h using the following concentrations: Activin A and MDK (both Merck, Darmstadt, Germany) 50 ng/ml, 100 ng/ml, 200 ng/ml; RBP4 (Abcam, Cambridge, UK) 10 µg/ml, 25 µg/ml, 50 µg/ml.

For determination of procollagen type I c-peptide concentration after treatment with secreted proteins, 13,000 fibroblasts/cm^2^ of five old donors (Table S1) were seeded in DMEM containing 10 % (v/v) FBS and 1 % (v/v) PS. Two days later FCS concentration was reduced to 2 %. After another 48 h secreted factors were diluted to final concentrations in DMEM containing 0.2 % (v/v) FBS and 1 % (v/v) PS and added to the fibroblasts.

For the analysis of proliferation 16,000 keratinocytes/cm^2^ of five old donors (Table S1) were seeded in KGM Gold. After 24 h secreted factors were added in KGM Gold.

## 3D skin equivalents

Nine 3D skin equivalents were generated: three that contained young fibroblast and six that contained old fibroblasts (Table S1). Three of the old 3D skin equivalents were subsequently used for Activin A-treatment. 3D skin equivalents were generated as previously described (Boehnke et al., 2007). Hyalograft-3D was replaced with M3-II Bemcot scaffold (Asahi Kasei, Tokyo, Japan). During the last 6 of 12 weeks culture, 100 ng/ml Activin A (Merck, Darmstadt, Germany) was added to the medium.

## Histology

Five sections of each 3D skin equivalent were generated at 6 µm thickness, mounted on slides and dried at room temperature for 1 h. Sections of 3D skin equivalents were stained with Diff-Quick (Labor + Technik Eberhard Lehmann, Berlin, Germany) according to manufacturer’s protocol and mounted with Entellan (Merck, Darmstadt, Germany). Slides were scanned using the Pannoramic SCAN II (3DHISTECH, Budapest, Hungary). Per slide a representative picture was taken in 20x magnification using case viewer software (3DHISTECH, Budapest, Hungary). Epidermal thickness was determined using Fiji software (Schindelin et al., 2012). A line was drawn at the interface of stratum granulosum and stratum corneum and a second line was drawn at the interface of stratum basale and the dermis. At a distance of 30 pixels, the distance between the two lines (the epidermal thickness) was determined.

## siRNA knockdown

One day prior transfection 8,000 fibroblasts/cm^2^ of five young donors (Table S1) were seeded. For the knockdown, fibroblasts were incubated with 10 nM siRNAs (Table S7), Lipofectamine® RNAiMAX and Opti-MEM Reduced-Serum Medium (Thermo Fisher Scientific Waltham, Massachusetts) for 24 h, according to manufacturer’s protocol. For the analysis of differentiation potential, knockdown was done in DMEM containing 10 % (v/v) FBS and 1 % (v/v) PS, for the analysis of proliferation, knockdown was done in DMEM containing 2 % (v/v) FBS and 1 % (v/v) PS. After another 48h, differentiation potential and/or proliferation were assessed.

## Analysis of differentiation potential

Three days after siRNA knockdown, differentiation potential was tested. Fibroblasts were shifted to adipogenic differentiation medium (Lonza, Basel, Switzerland) and cultured for two weeks. Fibroblasts were stained with 1:1000 HCS LipidTOX™ Deep Red Neutral Lipid Stain (Thermo Fisher Scientific, Waltham, Massachusetts) and 1:1000 Hoechst 33342 (Thermo Fisher Scientific, Waltham, Massachusetts). Per well 36 images were taken using a Scan R high content screening station (Olympus, Tokyo, Japan) with 20x magnification. Percentage of fibroblasts that accumulated triglycerides in lipid droplets were quantified using Scan R analysis software version 3.1.1.

Fibroblasts were shifted to chondogenic differentiation medium (Promocell, Heidelberg, Germany) and cultured for approx. two to three weeks. Time in days was measured until three-dimensional cartilage-like structure formed (Figure S7).

## Proliferation assay

72h after siRNA transfection of fibroblasts or after treatment of keratinocytes with secreted proteins colorimetric BrdU cell proliferation ELISA (Roche, Basel, Switzerland) was performed utilizing Tecan infinite M200 (Tecan, Männedorf, Switzerland) according to manufacturer’s instructions.

## Determination of procollagen type I c-peptide concentration

Procollagen type I c-peptide ELISA (Takara, Kyoto, Japan) of supernatants of 3D skin equivalents and of fibroblasts treated with secreted factors was done using and Tecan infinite M200 (Tecan, Männedorf, Switzerland) according to manufacturer’s protocol.

For normalization, cell number was determined using SpectraMax i3 MiniMax (Molecular Devices, San Jose, California). Therefore, nuclei were stained with 1:2000 EarlyTox Live Red dye (Molecular Devices, San Jose, California) in 80 % (v/v) DPBS and 20 % (v/v) ethanol.

## Statistics

Statistical analysis of the cell number comparison was done using the Fisher’s exact test and of the pluripotency score using unpaired Wilcoxon rank sum tests. For all other comparisons two-tailed un/paired t-test or RM one-way ANOVA with Dunnett multiple testing correction were used.

# Supplementary Figures

**
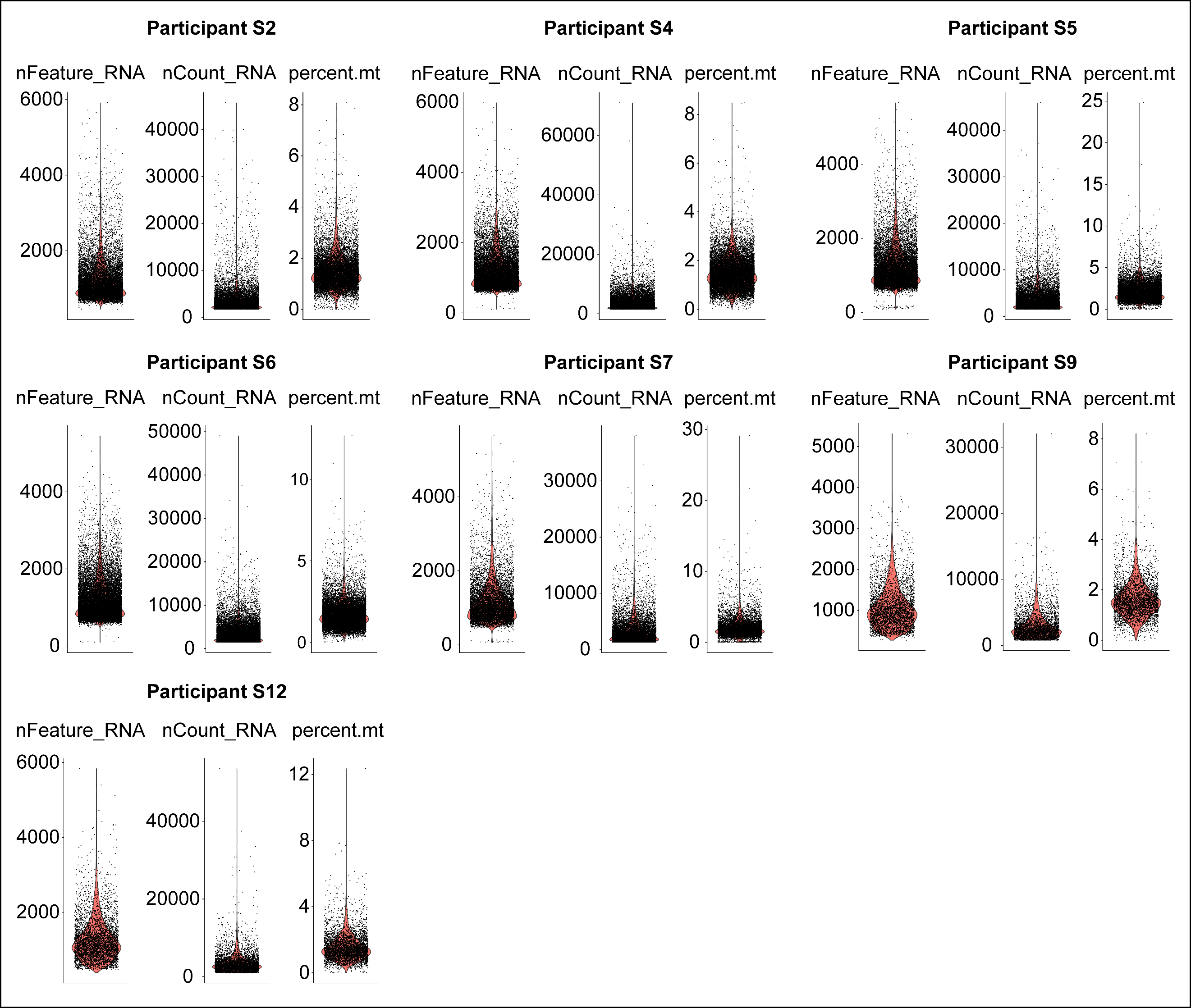
**

**Figure S1 Quality control metrics per participant.** Violin plots of quality control metrics for each study participant. Number of unique genes detected in each cell (nFeature_RNA), total number of molecules detected within a cell (nCount_RNA) and the percentage of reads that map to the mitochondrial genome (percent.mt).


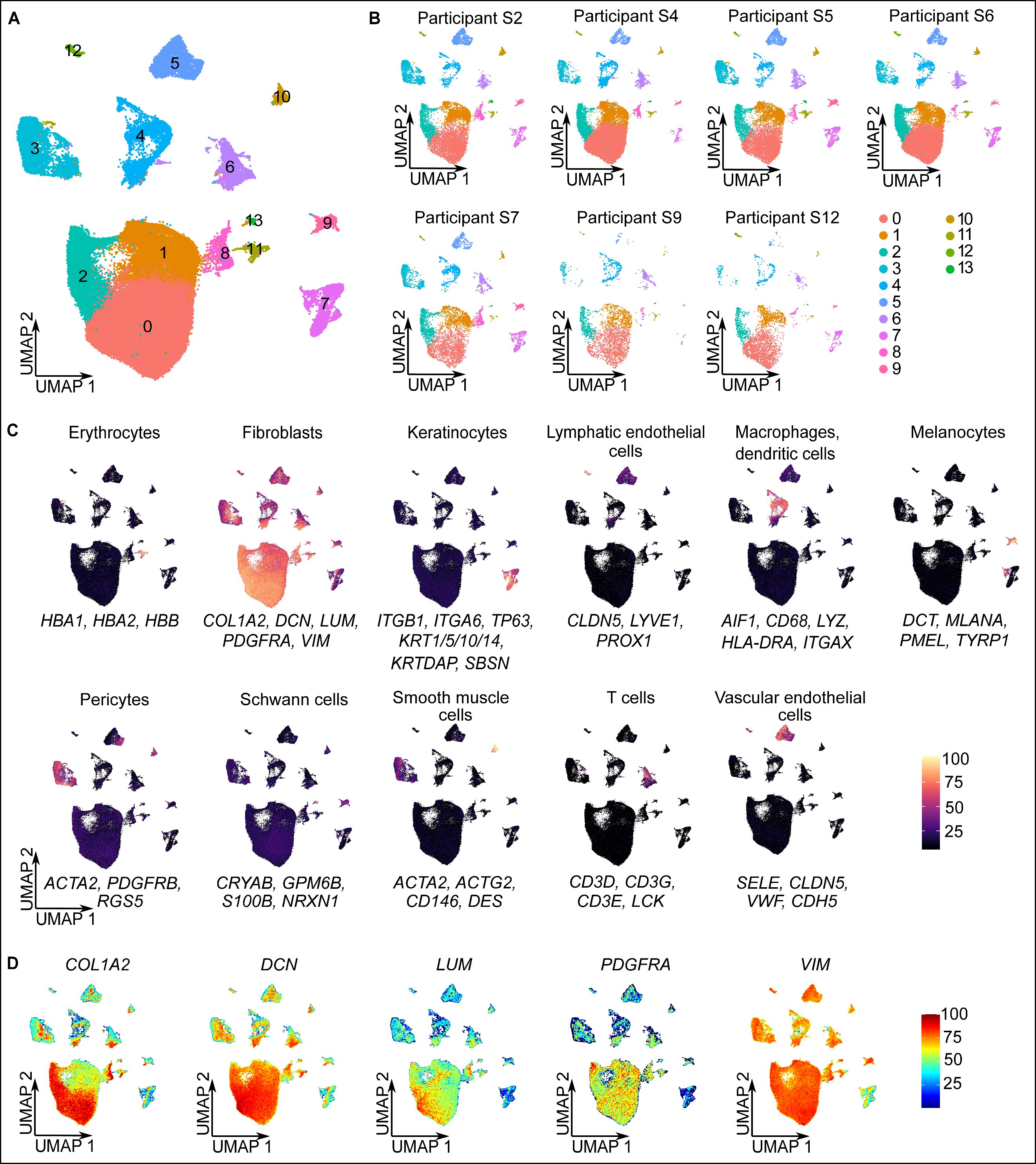


**Figure S2 14 clusters with distinct gene expression profiles represent 11 cell types.** (A) Uniform Manifold Approximation and Projection (UMAP) plot of 14 clusters of cells from all seven participants. (B) UMAP plots of 14 cell clusters for each study participant separately. (C) Average expression of well-established cell type markers among all cell types in UMAP plot. (D) Expression of markers used for the identification of fibroblasts among all cell types in UMAP plot.





**Figure S3 DS signature genes upregulated in subpopulation 4a.** Uniform Manifold Approximation and projection plots of (A) cell type clusters with a closeup of the DS population, the expression of the top 10 DS signature genes (Shin et al., 2020) and (B) the expression of *POSTN*, *PMEPA1* and *DPEP1*.





**Figure S4 Smooth muscle-related genes *ACTA2* and *TAGLN* upregulated in subpopulation 4a compared to other fibroblast populations.** Expression of smooth muscle-related genes (A) among fibroblast populations and (B) among all cell types in Uniform Manifold Approximation and projection (UMAP) plots. (C) Expression of additional muscle-related genes among all cell types in UMAP plots.


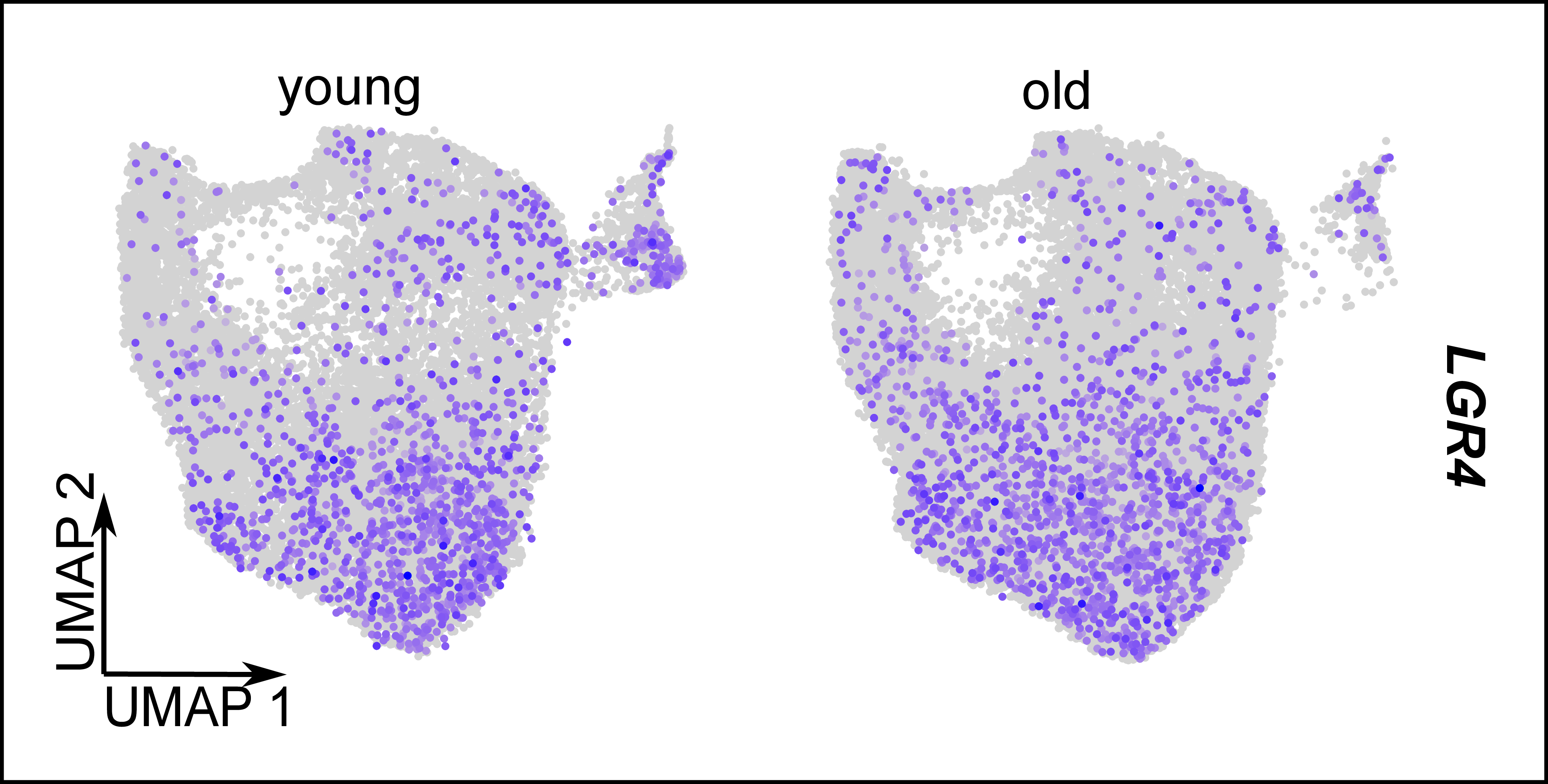


**Figure S5 Dermal sheath strongly expresses *LGR4* in young donors.** Fibroblasts expressing *LGR4* in blue in Uniform Manifold Approximation and Projection plots.

**Figure S6 Efficiency of siRNA knockdown on average >75 %.** Relative mRNA levels of genes in knockdown fibroblasts compared to control fibroblasts in percent.


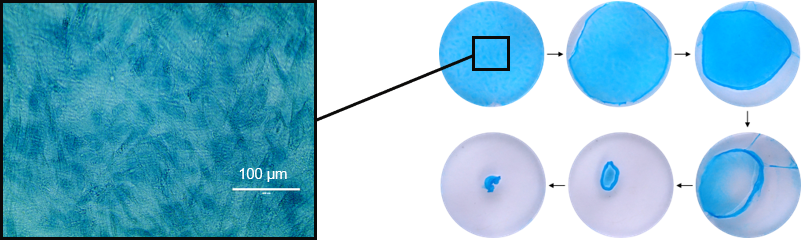


**Figure S7 Time course of chondrogenic differentiation.** Fibroblasts were cultured in chondrogenic differentiation medium until fibroblasts formed cartilage-like three-dimensional structures.

# Supplementary Tables

**Table S1 Overview of female Caucasian participants/ donors, skin samples and experiments, skin biopsies and isolated cells were used for.**

| \| **participant/**  **donor** \| **age** \| **body area** \| **experiment, passage** \| \| --- \| --- \| --- \| --- \| \| S2 \| 22 \| outer forearm \| single-cell RNA sequencing \| \| S4 \| 82 \| outer forearm \| single-cell RNA sequencing \| \| S5 \| 29 \| outer forearm \| single-cell RNA sequencing \| \| S6 \| 73 \| outer forearm \| single-cell RNA sequencing \| \| S7 \| 25 \| outer forearm \| single-cell RNA sequencing \| \| S9 \| 71 \| outer forearm \| single-cell RNA sequencing \| \| S12 \| 72 \| outer forearm \| single-cell RNA sequencing \| \| S003 \| 63 \| abdominal \| keratinocyte proliferation, p3 \| \| fibroblast procollagen synthesis, p4 \| \| S005 \| 66 \| abdominal \| keratinocyte proliferation, p3 \| \| fibroblast procollagen synthesis, p4 \| \| S007 \| 66 \| abdominal \| keratinocyte proliferation, p3 \| \| fibroblast procollagen synthesis, p4 \| \| S7 \| 65 \| abdominal \| keratinocyte proliferation, p3 \| \| fibroblast procollagen synthesis, p4 \| \| 63w4F16 \| 63 \| abdominal \| keratinocyte proliferation, p3 \| \| fibroblast procollagen synthesis, p5 \| \| St3 \| 22 \| abdominal \| siRNA knockdown experiments in fibroblasts, p4 \| \| St9 \| 27 \| breast \| siRNA knockdown experiments in fibroblasts, p4 \| \| St13 \| 27 \| breast \| siRNA knockdown experiments in fibroblasts, p4 \| \| St14 \| 29 \| breast \| siRNA knockdown experiments in fibroblasts, p4 \| \| St15 \| 19 \| breast \| siRNA knockdown experiments in fibroblasts, p4 \| \| 25wBa \| 25 \| abdominal \| fibroblasts for 3D skin equivalent, p4 \| \| 58wBa \| 58 \| abdominal \| fibroblasts for 3D skin equivalent, p4 \| \| 23w2K15 \| 23 \| breast \| keratinocytes for 3D skin equivalent, p3 \| \| 42wGe \| 42 \| face \| skin sections for RNA-FISH \| \| 50wGe \| 50 \| face \| skin sections RNA-FISH \| \| 77wSt \| 77 \| forehead \| skin sections RNA-FISH \| \| 70wGe \| 70 \| face \| skin sections RNA-FISH \| \| 60w \| 60 \| face \| skin sections RNA-FISH \| |  |  |  |  |
| --- | --- | --- | --- | --- | --- | --- | --- | --- | --- | --- | --- | --- | --- | --- | --- | --- | --- | --- | --- | --- | --- | --- | --- | --- | --- | --- | --- | --- | --- | --- | --- | --- | --- | --- | --- | --- | --- | --- | --- | --- | --- | --- | --- | --- | --- | --- | --- | --- | --- | --- | --- | --- | --- | --- | --- | --- | --- | --- | --- | --- | --- | --- | --- | --- | --- | --- | --- | --- | --- | --- | --- | --- | --- | --- | --- | --- | --- | --- | --- | --- | --- | --- | --- | --- | --- | --- | --- | --- | --- | --- | --- | --- | --- | --- | --- | --- | --- | --- | --- | --- | --- | --- | --- | --- | --- | --- | --- | --- | --- | --- | --- | --- | --- |

**Table S3 Differential gene expression between young and old subpopulation 4a cells.** Top 10 significantly (p-value < 0.05) upregulated genes in young versus old subpopulation 4a cells according to their log2 fold change (log2FC) identified by FindAllMarkers function.

| **log2FC** | **p_value** | **gene** |
| --- | --- | --- |
| 1.7739846 | 3.64E-22 | *COL11A1* |
| 1.5476803 | 6.63E-13 | *MYL4* |
| 1.4871236 | 2.17E-17 | *DPEP1* |
| 1.4768772 | 3.32E-11 | *TNMD* |
| 1.2038191 | 2.50E-06 | *FGFBP2* |
| 1.1888174 | 2.70E-08 | *WFDC1* |
| 1.1500604 | 7.85E-13 | *AMD1* |
| 1.1406357 | 2.78E-10 | *MEF2C* |
| 1.0084580 | 1.94E-08 | *TAGLN* |
| 0.9251109 | 0.00278081 | *C11orf96* |

**Table S5** **Representative expression values of candidate genes for siRNA knockdown in human dermal fibroblasts.** Gene, corresponding CT-value and 2^^(-deltaCT)^-value.

| **gene** | **CT-value** | **2^^(-deltaCT)^** |
| --- | --- | --- |
| *GAPDH* (internal control) | 18.04 |  |
| *SOX2* | 32.06 | 6 x 10^^-5^ |
| *HES1* | 27.16 | 1.8 x 10^^-3^ |
| *CTNNB1* | 21.40 | 0.097 |
| *DPEP1* | 33.81 | 1.8 x 10^^-5^ |
| *MYL4* | 32.55 | 4.3 x 10^^-5^ |
| *COL11A1* | 26.02 | 4.0 x 10^^-3^ |
| *SOX11* | 31.46 | 9.2 x 10^^-5^ |

**Table S6 Proteins secreted by dermal sheath population.** 25 secreted proteins with most significantly enriched expression in young dermal sheath population in comparison to the other fibroblast populations according to log2 fold change (log2FC). Potential signaling molecules underlined, available corresponding proteins specific for dermal sheath population in bold.

| **log2FC** | **p-value** | **gene** |
| --- | --- | --- |
| 2.330421 | 0 | *COL11A1* |
| 1.889525 | 3.78E-298 | *POSTN* |
| 1.339778 | 0 | *WFDC1* |
| 1.150726 | 2.83E-208 | ***MDK*** |
| 1.109674 | 1.75E-124 | *COL5A2* |
| 1.028636 | 7.67E-151 | *ASPN* |
| 0.898058 | 7.24E-104 | *COL15A1* |
| 0.888169 | 1.90E-59 | *PRSS23* |
| 0.875958 | 1.26E-79 | *COL6A3* |
| 0.816105 | 0 | *HAPLN1* |
| 0.768242 | 5.31E-130 | *COL4A1* |
| 0.7552721 | 3.63E-86 | *DKK3* |
| 0.753186 | 9.83E-227 | *EDIL3* |
| 0.751156 | 0 | *CFHR1* |
| 0.749335 | 1.82E-101 | *FRZB* |
| 0.740563 | 2.19E-125 | *CCDC3* |
| 0.735839 | 1.76E-66 | *SFRP1* |
| 0.724991 | 7.57E-53 | *COL1A1* |
| 0.696658 | 1.26E-107 | ***INHBA*** |
| 0.670393 | 1.88E-234 | *TNN* |
| 0.670393 | 5.35E-155 | ***RBP4*** |
| 0.656535 | 1.06E-99 | *LRRC17* |
| 0.583646 | 8.77E-166 | *COL21A1* |
| 0.575355 | 5.29E-65 | *COL4A2* |
| 0.574256 | 1.51E-89 | *EMID1* |

**Table S7 siRNAs used in knockdown experiments.**

| **Silencer Select siRNA** | **Manufacturer** | **ID** |  |
| --- | --- | --- | --- |
| *COL11A1* | Thermo Fisher Scientific, Waltham, Massachusetts | s533613 |  |
|  |  |  |  |
| *CTNNB1* | Thermo Fisher Scientific, Waltham, Massachusetts | s437 |  |
|  |  |  |  |
| *DPEP1* | Thermo Fisher Scientific, Waltham, Massachusetts | s4245 |  |
|  |  |  |  |
| *HES1* | Thermo Fisher Scientific, Waltham, Massachusetts | s6920 |  |
|  |  |  |  |
| *MYL4* | Thermo Fisher Scientific, Waltham, Massachusetts | s9185 |  |
|  |  |  |  |
| negative control #2 siRNA | Thermo Fisher Scientific, Waltham, Massachusetts | 4390846 |  |
|  |  |  |  |
| *SOX2* | Thermo Fisher Scientific, Waltham, Massachusetts | s13295 |  |
|  |  |  |  |
| *SOX11* | Thermo Fisher Scientific, Waltham, Massachusetts | s194809 |  |
|  |  |  |  |

**Table S8 Exclusion criteria for participation in the single-cell sequencing study.**

| **exclusion criteria** |
| --- |
| - Employee at the participating institutions (bioskin GmbH or Beiersdorf AG) |
| - Participation in another study involving the application of test products on the relevant area within two months before screening or during the study |
| - Male gender |
| - Aged < 18 or 30 - 65 years |
| - Body–Mass-Index (BMI) of ≤ 18 or ≥ 30 kg/m^2^ |
| - Pregnancy, planned pregnancy or nursing |
| - Nicotine abuse (more than 10 cigarettes per day) or alcohol abuse (more than 12 units alcohol per week, (one unit is equivalent to one glass of beer [ca. 330 ml] or one glass of wine [ca. 150 ml] or one high proof alcoholic drink [ca. 40 ml]), suspected drug abuse |
| - Radiation therapy within the last 2 years before screening |
| - Known allergy/ intolerance against prilocaine/other local anesthetics |
| - Intake of medications, which 1) show pronounced UV dependent reactions and/ or 2) may promote the development of wound infections (immune suppressive drugs), and/ or 3) may provoke a delay in wound healing medications (e.g. corticosteroids). Intake of blood thinning medications, of hormone preparation (e.g. menopausal complaints). |
| - Abnormal scar formation (e.g. hypertrophic scar formation/ keloid building) |
| - Clinically significant illness (e.g. infections, malignant diseases, skin diseases, diseases of internal organs e.g. diabetes mellitus, chronic inflammatory diseases or autoimmune diseases) that, in the opinion of the investigator 1) may jeopardize subject’s safety within the context of study conduct, and/or 2) may have an influence on the performance of the study and /or may influence the outcome and the results of the study |
| - Acute skin infections, Tattoos, suntan, eczema, hyperpigmentation or scars in the area of biopsy sampling or any other skin condition that in the opinion of the investigator may influence the conduct or the evaluation of the study |
| - Usage of self-tanner in the area of biopsy sampling within the last four weeks before screening |
| - Exposition to intense UV light (solarium/sunbathing) without the use of protective products with SPF 50 or higher in the four weeks before study begin |

# Supplementary references

Ashburner M, Ball CA, Blake JA, Botstein D, Butler H, Cherry JM, et al. Gene ontology: tool for the unification of biology. The Gene Ontology Consortium. Nat Genet 2000;25(1):25-9.

Bang ML, Vainshtein A, Yang HJ, Eshed-Eisenbach Y, Devaux J, Werner HB, et al. Glial M6B stabilizes the axonal membrane at peripheral nodes of Ranvier. Glia 2018;66(4):801-12.

Boehnke K, Mirancea N, Pavesio A, Fusenig NE, Boukamp P, Stark HJ. Effects of fibroblasts and microenvironment on epidermal regeneration and tissue function in long-term skin equivalents. Eur J Cell Biol 2007;86(11-12):731-46.

Castro R, Taetzsch T, Vaughan SK, Godbe K, Chappell J, Settlage RE, et al. Specific labeling of synaptic schwann cells reveals unique cellular and molecular features. Elife 2020;9.

Gene Ontology C. The Gene Ontology resource: enriching a GOld mine. Nucleic Acids Res 2021;49(D1):D325-D34.

Halim D, Hofstra RM, Signorile L, Verdijk RM, van der Werf CS, Sribudiani Y, et al. ACTG2 variants impair actin polymerization in sporadic Megacystis Microcolon Intestinal Hypoperistalsis Syndrome. Hum Mol Genet 2016;25(3):571-83.

Hao Y, Hao S, Andersen-Nissen E, MauckIII WM, Zheng S, Butler A, et al. Integrated analysis of multimodal single-cell data. bioRxiv 2020.

Kilpinen H, Goncalves A, Leha A, Afzal V, Alasoo K, Ashford S, et al. Transcription profiling by array of human fibroblasts and induced pluripotent stem cells (iPS cells) from the skin of heathy volunteers as part of the HipSci project. Array Express; 2017, E-MTAB-4057.

La Manno G, Soldatov R, Zeisel A, Braun E, Hochgerner H, Petukhov V, et al. RNA velocity of single cells. Nature 2018;560(7719):494-8.

Lawrence M, Gentleman R, Carey V. rtracklayer: an R package for interfacing with genome browsers. Bioinformatics 2009;25(14):1841-2.

Li H. A statistical framework for SNP calling, mutation discovery, association mapping and population genetical parameter estimation from sequencing data. Bioinformatics 2011;27(21):2987-93.

Li H, Handsaker B, Wysoker A, Fennell T, Ruan J, Homer N, et al. The Sequence Alignment/Map format and SAMtools. Bioinformatics 2009;25(16):2078-9.

Lim EF, Nakanishi ST, Hoghooghi V, Eaton SE, Palmer AL, Frederick A, et al. AlphaB-crystallin regulates remyelination after peripheral nerve injury. Proc Natl Acad Sci U S A 2017;114(9):E1707-E16.

Mata M, Alessi D, Fink DJ. S100 is preferentially distributed in myelin-forming Schwann cells. J Neurocytol 1990;19(3):432-42.

Mi H, Muruganujan A, Ebert D, Huang X, Thomas PD. PANTHER version 14: more genomes, a new PANTHER GO-slim and improvements in enrichment analysis tools. Nucleic Acids Res 2019;47(D1):D419-D26.

Paulin D, Li Z. Desmin: a major intermediate filament protein essential for the structural integrity and function of muscle. Exp Cell Res 2004;301(1):1-7.

R Core Team. R: A language and environment for statistical computing. Vienna, Austria: R Foundation for Statistical Computing; 2020, <https://www.R-project.org/>.

Rojahn T, Vorstandlechner V, T K, Bauer W, Alkon N, Bangert C, et al. Single-cell Transcriptomics Combined With Interstitial Fluid Proteomics Defines Cell Type-Specific Immune Regulation in Atopic Dermatitis. Gene Expression Omnibus; 2020, GSE153760.

Roostalu U, Aldeiri B, Albertini A, Humphreys N, Simonsen-Jackson M, Wong JKF, et al. Distinct Cellular Mechanisms Underlie Smooth Muscle Turnover in Vascular Development and Repair. Circ Res 2018;122(2):267-81.

Schindelin J, Arganda-Carreras I, Frise E, Kaynig V, Longair M, Pietzsch T, et al. Fiji: an open-source platform for biological-image analysis. Nat Methods 2012;9(7):676-82.

Shin W, Rosin NL, Sparks H, Sinha S, Rahmani W, Sharma N, et al. Dysfunction of Hair Follicle Mesenchymal Progenitors Contributes to Age-Associated Hair Loss. Dev Cell 2020;53(2):185-98 e7.

Smit AFA, Hubley R, Green P. RepeatMasker Open-4.0. 2013-2015.

Solé-Boldo L, Günter Raddatz G, Schütz S, Mallm J, Rippe K, Lonsdorf A, et al. Single-cell transcriptomes of the aging human skin reveal loss of fibroblast priming. Gene Expression Omnibus; 2020, GSE130973.

Sole-Boldo L, Raddatz G, Schutz S, Mallm JP, Rippe K, Lonsdorf AS, et al. Single-cell transcriptomes of the human skin reveal age-related loss of fibroblast priming. Commun Biol 2020;3(1):188.

Stettner M, Zenker J, Klingler F, Szepanowski F, Hartung HP, Mausberg AK, et al. The Role of Peripheral Myelin Protein 2 in Remyelination. Cell Mol Neurobiol 2018;38(2):487-96.

Stuart T, Butler A, Hoffman P, Hafemeister C, Papalexi E, Mauck WM, 3rd, et al. Comprehensive Integration of Single-Cell Data. Cell 2019;177(7):1888-902 e21.

VanRossum G, Drake FL. Python 3 Reference Manual. Scotts Valley, CA: CreateSpace; 2009.

Wickham H. ggplot2: Elegant Graphics for Data Analysis. New York: Springer-Verlag; 2016, <https://ggplot2.tidyverse.org>.

Yates AD, Achuthan P, Akanni W, Allen J, Allen J, Alvarez-Jarreta J, et al. Ensembl 2020. Nucleic Acids Res 2020;48(D1):D682-D8.

Yuan SM. alpha-Smooth Muscle Actin and ACTA2 Gene Expressions in Vasculopathies. Braz J Cardiovasc Surg 2015;30(6):644-9.

Zheng GX, Terry JM, Belgrader P, Ryvkin P, Bent ZW, Wilson R, et al. Massively parallel digital transcriptional profiling of single cells. Nat Commun 2017;8:14049.
